# A Novel Therapeutic Approach: Phage and *Alhagi maurorum* Synergy Against Kidney and Bladder Infection Caused by *Proteus mirabilis* in BALB/c Mice

**DOI:** 10.1155/sci5/2249075

**Published:** 2026-08-25

**Authors:** Sharareh Moghim, Samira Choopani, Bahram Nasr Esfahani, Jean-Paul Pirnay, Jeroen Wagemans, Rob Lavigne, Arezoo Mirzaei

**Affiliations:** ^1^ Department of Bacteriology and Virology, School of Medicine, Isfahan University of Medical Sciences, Isfahan, Iran, mui.ac.ir; ^2^ Department of Physiology, School of Medicine, Isfahan University of Medical Sciences, Isfahan, Iran, mui.ac.ir; ^3^ Laboratory for Molecular and Cellular Technology, Queen Astrid Military Hospital, Brussels, Belgium; ^4^ Department of Biosystems, KU Leuven, Leuven, Belgium, kuleuven.be

**Keywords:** *Alhagi maurorum*, bacteriophages, *Proteus mirabilis*, urologic diseases

## Abstract

Catheter‐associated urinary tract infections (CAUTIs) caused by *Proteus mirabilis* biofilms present significant challenges due to escalating multidrug resistance, highlighting the urgent need for alternative therapeutic strategies. This study evaluates a novel combinatorial approach employing a cocktail of two lytic bacteriophages (Isf‐Pm1 and Isf‐Pm2) in conjunction with *Alhagi maurorum* ethanolic extract (AME) to reduce bacterial burden in an established murine model of catheter‐associated infection. We hypothesized that the synergistic activity of phages and AME would enhance treatment efficacy, impede resistance development, and promote infection clearance. Using a catheterized BALB/c mouse model with five experimental groups (*n* = 6 per group), we assessed the therapeutic efficacy of the phage–AME combination across varying dosages and treatment durations. Bacterial load (CFU/mL) was quantified from homogenized kidney and bladder tissues following treatment. The combination of phages at a multiplicity of infection (MOI) of 1 and AME at 750 mg/kg administered over 7 days produced the most pronounced antibacterial effect, achieving a 7‐log_10_ reduction in renal bacterial counts and a 5.3‐log_10_ reduction in bladder bacterial counts. Notably, extending the treatment duration from 3 to 7 days significantly enhanced bacterial clearance in renal tissues (*p* < 0.0001), highlighting the critical importance of prolonged therapy for optimizing outcomes. Our findings demonstrate that both dosage and treatment duration significantly influence the therapeutic efficacy of the phage–AME combination, emphasizing the necessity of protocol optimization to improve clinical outcomes for patients with kidney and bladder infections. Collectively, this study presents a promising, mechanistically grounded alternative strategy for combating biofilm‐associated CAUTIs and warrants further translational investigation.

## 1. Introduction

Catheter‐associated urinary tract infections (CAUTIs) represent a significant clinical challenge, with *Proteus mirabilis* identified as one of the predominant uropathogens associated with these infections [1]. The ability of *P. mirabilis* to form robust biofilms on catheter surfaces complicates treatment, as these biofilms create a protective niche that renders bacteria highly resistant to conventional antibiotic therapies. Consequently, there is an urgent need for alternative therapeutic strategies to address *P. mirabilis*‐associated CAUTIs [2].

Phage therapy has emerged as a promising strategy for managing bacterial infections, particularly those characterized by biofilm formation [3]. Bacteriophages are naturally occurring viruses that specifically target and infect bacterial cells, enabling selective eradication of pathogenic bacteria while preserving beneficial microbes [4].

Although numerous studies have demonstrated the effectiveness of phage therapy in reducing bacterial load and disrupting biofilms in vitro and in animal models [5–7], its specific application against *P. mirabilis* biofilms in vivo remains relatively underexplored.

An alternative approach involves phytochemical therapy. *Alhagi maurorum* ethanolic extract (AME) has shown promise as an adjunctive treatment for CAUTIs [8]. Its bioactive constituents, including flavonoids and alkaloids, exhibit antimicrobial activity against common uropathogens, including *Escherichia coli* and *P. mirabilis* [8, 9].

Notably, *A. maurorum* metabolites demonstrate quorum‐quenching activity by suppressing the *P. mirabilis lux*S‐R system and inhibiting biofilm maturation in vitro, suggesting their utility as virulence attenuators [8, 10].

Theoretically, combining phages with phytochemical agents such as AME offers a dual‐mode strategy against CAUTIs; AME attenuates virulence and weakens biofilm architecture (thereby reducing bacterial defenses and quorum sensing), while phages provide targeted bacteriolysis and penetrate deep into biofilms. This synergistic approach may also reduce selective pressure for resistance compared with monotherapy, thereby improving overall therapeutic efficacy in biofilm‐associated infections [11–13]. Despite growing interest in phage therapy and phytochemicals as separate interventions, their combined use has not been well explored in vivo.

This study aims to evaluate the efficacy of phage therapy in combination with AME in reducing *P. mirabilis* burden in a catheterized BALB/c mouse model of CAUTI. We assess the impact of treatment on bacterial load and infection severity, contributing to the expanding body of knowledge on combination strategies for biofilm‐related infections. To our knowledge, this is one of the first in vivo investigations to evaluate the combined effects of bacteriophages and *A. maurorum* extract against *P. mirabilis* CAUTIs, thereby helping to bridge the translational gap between encouraging in vitro findings and animal‐model validation. The outcomes of this investigation may facilitate the development of innovative therapeutic strategies to address the pressing need for alternatives to conventional antibiotics.

## 2. Materials and Methods

### 2.1. Ethical Approval and Animal Housing

The study was conducted at Isfahan University of Medical Sciences in compliance with international guidelines for animal research and approved by the local ethical committee (permit number: IR.MUI.AEC.1401.045). Female BALB/c mice (6–8 weeks old, 20–22 g) were obtained from the Royan Institute breeding facility (Isfahan, Iran). The mice were housed in a controlled environment with a 12‐h light/dark cycle at 21°C and given food and water to ensure their well‐being throughout the study.

### 2.2. Bacteriophage Propagation, Hydroalcoholic Extraction of *Alhagi maurorum*

The previously characterized double‐stranded DNA phages, Isf‐Pm1 and Isf‐Pm2 (with a latent period of 60 min and estimated burst sizes of 46 and 666 phage particles per infected bacterium, respectively) [14], which were stored in −70°C, were propagated in 2 × Lysogeny Broth (LB) supplemented with 1 mM CaCl_2_ using *P. mirabilis* ATCC 7002 (1.5 × 10^8^ CFU/mL) at 37°C with shaking for 16 h. Lysates were centrifuged (10, 000 × g, 15 min, 4°C), filtered (0.22 μm), and phage presence was confirmed by spot testing. Titers were determined by the double‐layer agar assay as outlined by Andre Gratia and expressed as PFU/mL [15]. For in vivo use, equal volumes of both phages were combined to prepare a cocktail at 1.0 × 10^8^ PFU/mL, and MOI was calculated relative to the initial bacterial inoculum.

The extract previously prepared by the hydroalcoholic method and stored at −20°C was suspended in distilled water to achieve the desired concentrations (500 and 750 mg/kg body weight) [8]. The doses of AME (500 and 750 mg/kg) were selected based on previous in vitro efficacy data and on published in vivo studies using comparable extracts and administration routes [8, 16].

### 2.3. Animal Treatment

**FIGURE 1:**
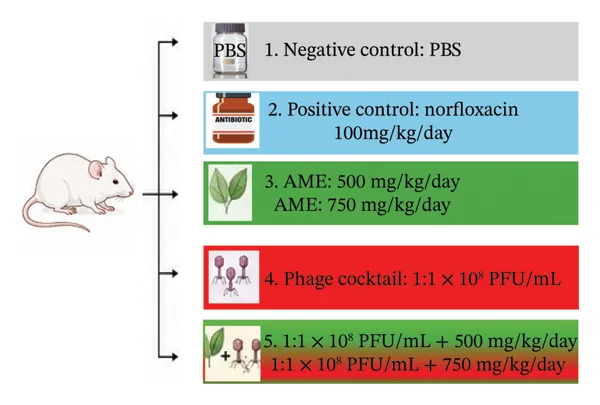
Experimental design and group assignment.

A randomized, partially blinded study was designed comprising five groups of BALB/c mice (*n* = 6 per group); a PBS mock control, a norfloxacin‐positive control (100 mg/kg/day), and three test groups receiving AME (500 or 750 mg/kg/day), a phage cocktail (1.0 × 10^8^ PFU/mL, MOI 1), or their combination. Mice were randomly assigned to groups to ensure unbiased allocation (Figure 1).

**FIGURE 2:**
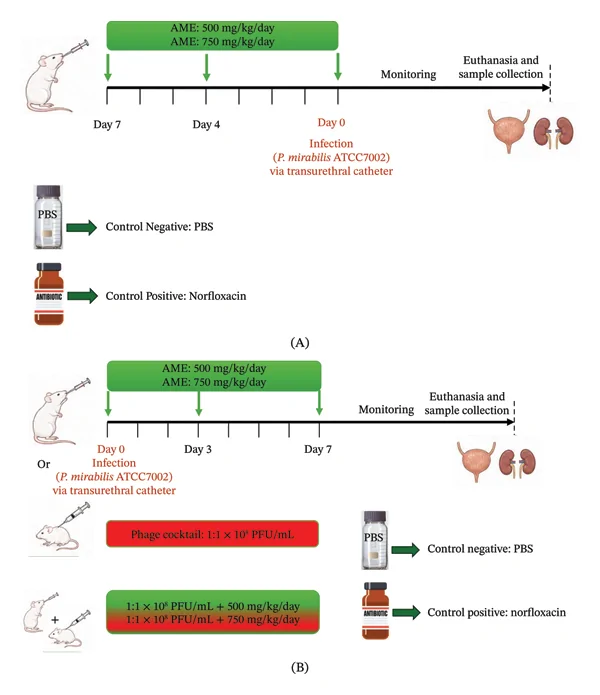
Pretreatment and posttreatment experimental schedules. Mice were assigned to pretreatment or posttreatment protocols. (A) In the pretreatment schedule, AME was administered for 4 or 7 days before catheter‐induced infection with *P. mirabilis* ATCC 7002. (B) In the posttreatment schedule, treatment began 6 h after infection and continued for 3 or 7 days. Treatments included AME (500 or 750 mg/kg/day), a phage cocktail (MOI = 1), their combination, norfloxacin as the positive control, and PBS as the negative control.

To evaluate prophylactic and therapeutic effects, two schedules were applied. In the pretreatment schedule, AME was administered by intragastric gavage (20‐gauge feeding needle) for 4 or 7 days prior to infection. In the posttreatment schedule, treatment was initiated 6 h postinfection and continued for 3 or 7 days. All treatments were delivered in 500‐μL volumes; AME was freshly dissolved in distilled water [8], and norfloxacin and PBS served as positive and negative controls, respectively (Figure 2).

Infection was established following Lane et al. [17]. Mice were anesthetized with ketamine/xylazine (70/5 mg/kg) and transurethrally inoculated with 20 μL of *P. mirabilis* ATCC 7002 (1.5 × 10^8^ CFU) via a sterile PE‐10 catheter. At termination, animals were euthanized by cervical dislocation; bladders and kidneys were aseptically excised, homogenized in 1‐mL PBS (8000 rpm, 10 s, × 3), and spiral‐plated on LB agar for 24 h at 37°C. Bacterial loads were quantified as CFU/mL of homogenate and expressed per gram of tissue.

### 2.4. Statistical Analysis

Statistical analysis was performed by GraphPad Prism (Version 9). Two‐way ANOVA was used to assess the main effects of the two independent factors and their interaction; when significant differences were detected, Tukey’s post hoc multiple comparisons test was performed as complementary tests. Statistical significance was set at *p* < 0.05.

## 3. Results

### 3.1. AME Pretreatment

To evaluate the prophylactic effect of AME, mice were pretreated with the extract at 500 or 750 mg/kg/day for 4 or 7 days, respectively, prior to bacterial challenge with *P. mirabilis* ATCC 7002. The bacterial load in the kidneys and bladders was subsequently quantified to assess protection.

**FIGURE 3:**
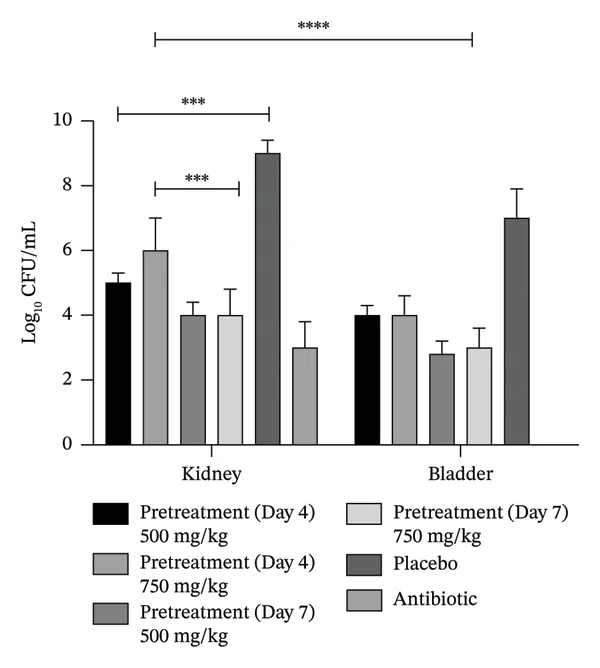
Prophylactic effect of AME pretreatment on renal and bladder bacterial loads following *P. mirabilis* challenge. Mice received AME at 500 or 750 mg/kg/day for 4 or 7 days prior to infection with *P. mirabilis* ATCC 7002. Bacterial loads in kidneys and bladders were quantified 24 h after challenge. AME pretreatment lowered renal bacterial burden compared with the placebo group at both concentrations. A 7‐day pretreatment period (500 or 750 mg/kg) produced a greater reduction in kidney bacterial load than the 4‐day 750 mg/kg regimen, showing that duration had a stronger effect than dose. In the bladder, all AME regimens produced consistent and marked reductions in bacterial load relative to placebo, indicating a more responsive prophylactic effect in this tissue. Error bars represent standard deviation. Significance levels: ^∗∗∗^ *p* = 0.0003, ^∗∗∗∗^ *p* < 0.0001.

Pretreatment with AME significantly reduced renal bacterial load compared with the placebo group at both tested concentrations (Figure 3). Furthermore, the analysis revealed that treatment duration was a critical factor for renal protection. Specifically, pretreatment for 7 days (at either 500 or 750 mg/kg) resulted in a significantly lower kidney bacterial load than the 4‐day regimen at 750 mg/kg. This suggests that a longer pretreatment duration is more critical for renal protection than a higher dose over a shorter period.

In contrast, all AME pretreatment regimens, regardless of dose or duration, demonstrated a more pronounced and uniformly successful reduction of bacterial load in the bladder compared to the kidney, indicating a tissue‐specific prophylactic effect.

### 3.2. AME Posttreatment

**FIGURE 4:**
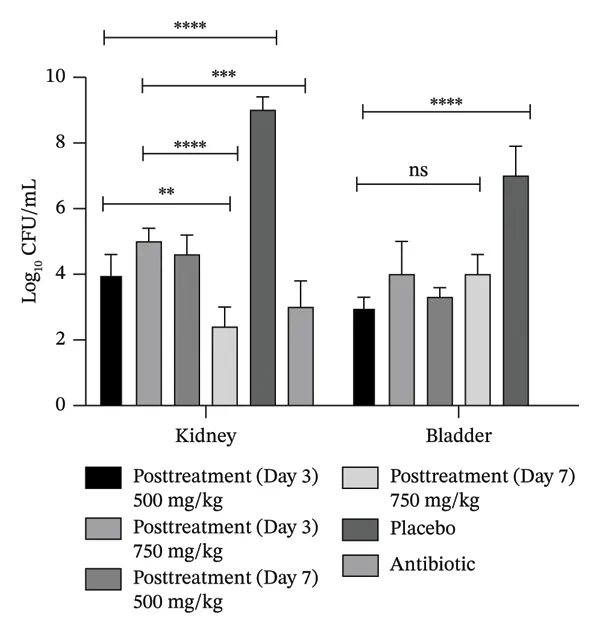
Posttreatment efficacy of AME against *P. mirabilis* infection in the kidneys and bladder. Mice infected with *P. mirabilis* ATCC 7002 received AME at 500 or 750 mg/kg/day for 3 or 7 days, and bacterial loads were measured in the kidneys and bladder. All AME regimens significantly lowered kidney bacterial burden relative to placebo, with the 750 mg/kg 7‐day treatment producing the greatest reduction. Longer treatment improved renal outcomes more than a higher dose alone. Comparisons with the antibiotic control showed that norfloxacin outperformed several AME regimens. In the bladder, all AME treatments reduced bacterial load compared with placebo, though none matched the antibiotic. No significant differences were observed among AME doses or durations in bladder tissue. Error bars indicate standard deviation; significance levels: ^∗∗^ *p* = 0.008,^∗∗∗^ *p* = 0.0002, ^∗∗∗∗^ *p* < 0.0001, ns = not significant.

Posttreatment with AME reduced bacterial burden in both kidneys and the bladder, with effects influenced by dose and treatment duration (Figure 4). In the kidney, all AME regimens significantly reduced bacterial load compared with placebo (*p* < 0.0001). The 750 mg/kg regimen administered for 7 days produced the greatest reduction and was significantly more effective than shorter or lower dose regimens. Norfloxacin remained more effective than the less intensive AME schedules, although the 750 mg/kg for 7‐day regimen approached the antibiotic effect more closely than the other AME treatments.

In the bladder, all AME regimens also significantly decreased bacterial load relative to placebo (*p* < 0.0001); however, none achieved the same level of efficacy as norfloxacin (*p* < 0.0001). No significant differences were observed among AME doses or durations in the bladder. Overall, the 750 mg/kg for 7‐day regimen was the most effective AME posttreatment, particularly in the kidney.

### 3.3. Phage Posttreatment

**FIGURE 5:**
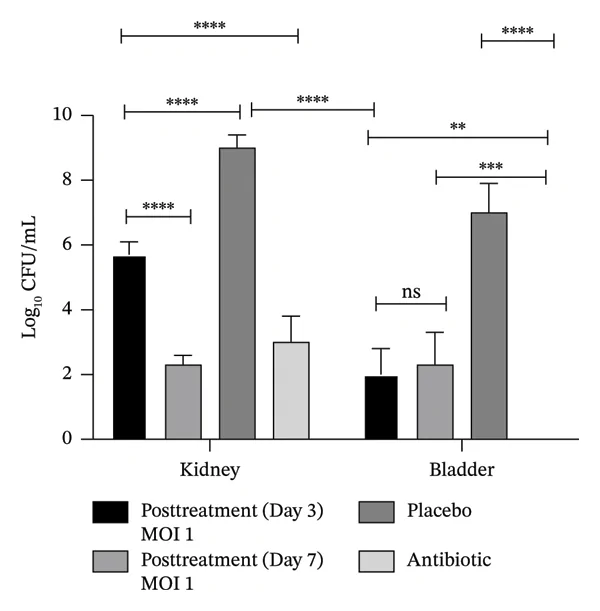
Posttreatment efficacy of phage therapy (MOI 1) in the kidneys and bladder following *P. mirabilis* infection. Mice received phage therapy at MOI 1 for either 3 or 7 days after infection. Both regimens significantly lowered kidney bacterial loads compared with the placebo group, and the 7‐day course produced a markedly stronger effect than the 3‐day treatment. The shorter regimen remained less effective than the antibiotic control, whereas the 7‐day phage treatment matched the antibiotic with no detectable difference. Tissue responses diverged: the bladder showed a faster decline in bacterial burden, with the 3‐day phage treatment yielding lower counts than those seen in the kidney after 7 days. By Day 7, kidney loads reached levels similar to those in the bladder at both time points. Error bars represent standard deviation; ns = not significant; ^∗∗^ *p* = 0.0011, ^∗∗∗^ *p* = 0.001, ^∗∗∗∗^ *p* < 0.0001.

We evaluated the effectiveness of the optimal phage therapy (MOI of 1) after 3‐day and 7‐day courses. The results showed that a full 7 days of treatment was significantly more effective at clearing the kidney infection than just 3 days (*p* < 0.0001), establishing treatment duration as a critical factor for success (Figure 5). Furthermore, phage therapy at both time points significantly reduced bacterial load in the kidneys compared with placebo (*p* < 0.0001).

Compared with the antibiotic control (norfloxacin), the 3‐day phage regimen was significantly less effective in the kidneys (*p* < 0.0001). However, this difference disappeared after 7 days of phage treatment, which performed as well as the antibiotic, with no statistically significant difference between them.

The response to phage therapy also differed by organ. After 3 days, treatment was significantly more effective in the bladder than in the kidney (*p* < 0.0001). In fact, the bacterial load in the bladder after just 3 days was even lower than that in the kidney after a full 7 days, highlighting the bladder’s more rapid and robust response. Finally, we found that the bacterial load in the kidneys after 7 days of phage therapy was not significantly different from the levels found in the bladder after either 3‐ or 7‐day treatment regimen (Figure 5).

### 3.4. Combination Posttreatment

**FIGURE 6:**
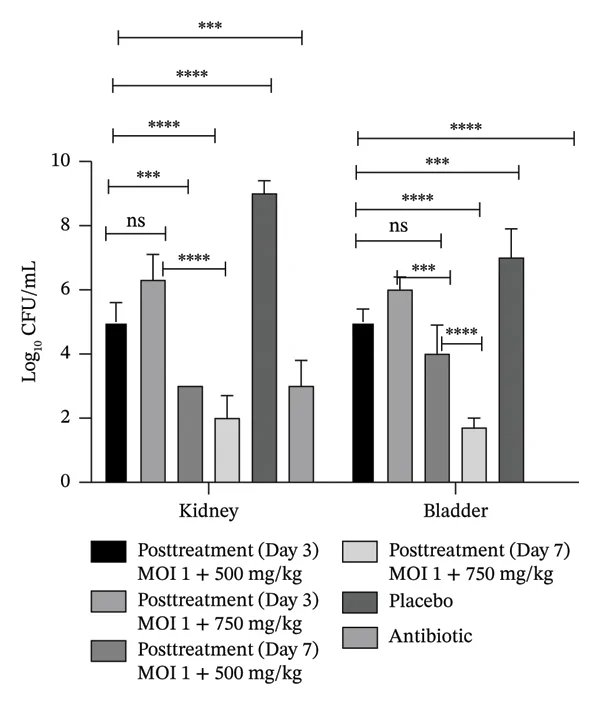
Posttreatment effects of combined phage therapy (MOI 1) and AME on kidney and bladder bacterial loads after *P. mirabilis* infection. Mice were treated with phage (MOI 1) together with AME at 500 or 750 mg/kg for 3 or 7 days. Combination therapy significantly lowered bacterial loads in both organs compared with placebo across all effective regimens. In the kidneys, extended treatment (7 days) consistently outperformed the 3‐day courses, and the 750 mg/kg 7‐day regimen produced the strongest reduction. Dose effects appeared only when treatment lasted 7 days. In the bladder, longer treatment similarly improved outcomes, and all 7‐day regimens reduced bacterial burdens far more than placebo. Several comparisons showed no significant difference between the most effective combination treatments and phage alone at 7 days, and no differences emerged between the kidney and bladder when both received AME 750 mg/kg with MOI 1 for 7 days. Error bars show standard deviation; ns = not significant; ^∗∗∗^ *p* = 0.0002, ^∗∗∗∗^ *p* < 0.0001.

**FIGURE 7:**
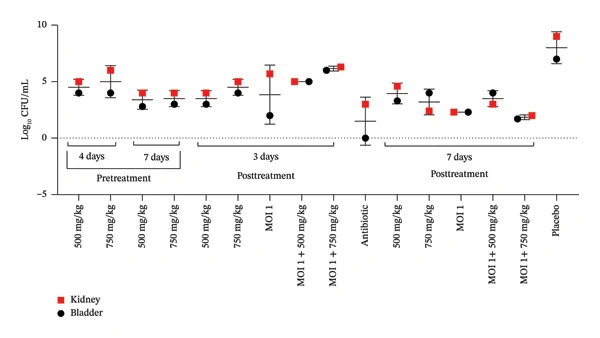
Scatter plot of pretreatment and posttreatment effects of AME, phage therapy, and combination regimens on kidney and bladder bacterial loads. Bacterial burdens (Log_10_ CFU/mL) were measured in the kidneys and bladders of mice following *P. mirabilis* infection. Pretreatment groups received AME at 500 or 750 mg/kg for either 4 or 7 days before bacterial challenge. Posttreatment groups received AME alone, phage therapy (MOI 1), or combined AME–phage regimens for 3 or 7 days after infection. Across most conditions, longer treatment durations produced greater reductions in bacterial load than higher AME doses alone. Combination treatments and extended regimens showed the strongest decreases, particularly in the kidney. The antibiotic group had one of the lowest bacterial loads, while the placebo group had the highest levels. Square and circular symbols denoted kidney and bladder treatments, respectively. Error bars represent standard deviation.

Combination therapy with phages at MOI 1 and AME at 500 or 750 mg/kg was assessed after 3 and 7 days (Figures 6 and 7). In both the kidney and the bladder, the combination treatment significantly reduced bacterial loads compared with placebo, with efficacy increasing as both dose and duration increased. The most effective regimen, MOI 1 plus AME at 750 mg/kg for 7 days, produced a 7‐log reduction in kidney and a 5.3‐log reduction in bladder counts.

In the kidney, the 500 mg/kg for 3‐day regimen differed significantly from the 500 mg/kg for 7 days and 750 mg/kg for 7‐day regimens (*p* = 0.0002 and *p* < 0.0001, respectively), while the 750 mg/kg for 3‐day regimen also differed from the 750 mg/kg for 7‐day regimen (*p* < 0.0001). All kidney combination groups were significantly lower than placebo (*p* < 0.0001), and the optimal regimen showed no significant difference from norfloxacin. In the bladder, a similar pattern was observed, although norfloxacin remained significantly more effective than the combination groups, including the optimal regimen (*p* = 0.003) and the remaining regimens (*p* < 0.0001).

## 4. Discussion

*P. mirabilis* poses a considerable threat to human health as a frequent cause of CAUTI and hospital‐acquired infections. Confronting its escalating antibiotic resistance demands innovative therapeutic solutions. In response, research is advancing along two promising avenues: refined bacteriophage therapies, often used in synergistic combinations to circumvent resistance, and the application of plant‐derived essential oils and extracts. These natural compounds, rich in bioactive phytochemicals, effectively disrupt critical bacterial processes such as biofilm formation, offering a potent defense against *P. mirabilis* and other resilient pathogens [8, 11, 18–24].

Although the therapeutic value of phage cocktails and plant‐derived extracts is widely recognized, we still know surprisingly little about how these treatments might work together, especially when facing multidrug‐resistant pathogens. This gap opens a critical line of inquiry, particularly in our region, where medicinal plants such as *A. maurorum* have a long history of use in supporting kidney and renal function [8, 25, 26]. What makes this plant especially interesting is that it does not kill *P. mirabilis* outright; instead, it interferes with the bacterium’s signaling system, essentially disarming it without applying direct lethal pressure [8].

Building on this observation, combining this putative quorum‐quenching activity with phage‐mediated killing may enhance antibacterial efficacy while also helping to reduce biofilm‐associated persistence.

Furthermore, in vitro studies have indicated that this combined approach may enhance antibiofilm activity [11]. However, these findings must be further validated through in vivo studies, as the complexity of signaling pathways and host–pathogen interactions in animal models differs significantly from that observed in vitro.

Here, we combined both phage and AME treatment to investigate their potential synergy. The results of this study highlight significant differences in treatment outcomes across various kidney and bladder groups, providing insights into the efficacy of MOI 1 and AME combinations at different dosages and durations.

The significant increases in bacterial load observed in the kidney group treated with the combination at 500 mg/kg for 3 days, compared with both 500 mg/kg for 7 days and 750 mg/kg for 7 days, suggest that a shorter duration at a lower dose may yield the bacteria in specific contexts and not be effective as much as the other treatment with higher dose and longer duration. One possible explanation is that longer exposure and higher AME dose allowed greater cumulative antibacterial activity, which may have improved the overall effect of the combination treatment. This may have facilitated more effective phage‐mediated bacterial clearance.

This counterintuitive finding emphasizes the importance of treatment duration in therapeutic efficacy. Additionally, the significant decrease in mean bacterial load when comparing the 500 mg/kg for 3‐day group with the placebo group indicates that the treatment provided a beneficial effect compared to no treatment, reinforcing the importance of the treatment regimen.

The results also reveal that the kidney group, when treated with 750 mg/kg for 3 days, showed significant increases compared to the 500 mg/kg for 7‐day group. This further suggests that higher doses can lead to improved outcomes but may also depend on treatment duration, as evidenced by the significant decreases compared with the placebo group.

The consistent, significant decreases in groups treated with the combination at higher doses and longer durations (750 mg/kg for 7 days) compared to placebo and other treatments highlight the potential for these treatments to yield substantial benefits in kidney function.

In the bladder assessments, the significant increases observed in the bladder group at 500 mg/kg for 3 days, compared with 750 mg/kg for 7 days, suggest that lower dosages for shorter durations may effectively enhance bladder function. This parallels findings in the kidney assessments, indicating that treatment duration and dosage require careful consideration.

The significant decrease observed when comparing the 500 mg/kg for 3‐day group with the placebo underscores the treatment’s effectiveness, suggesting that even lower dosages can provide beneficial outcomes compared to inaction. This improvement may reflect interference with virulence‐associated processes such as quorum sensing and biofilm formation, although this was not directly examined in the present study.

In the bladder, treatment efficacy may have been influenced by physiological urine flow, which could reduce the local retention time of the administered agents. Overall, the treatment for kidney infections was more effective, and the combination of phages at an MOI of 1 and AME at 750 mg/kg body weight was the most effective compound for treating kidney infections. This difference may be related to longer exposure time and improved local effect in the kidney. AME and phage may have acted through complementary mechanisms that collectively improved bacterial clearance. In the bladder, continuous urine flow may have limited drug retention and reduced the opportunity for sustained combined activity.

The present findings indicate that the therapeutic response differed between the bladder and kidney‐treated groups, reflecting the distinct biological characteristics of these two anatomical sites. In the bladder‐treated groups, norfloxacin demonstrated a statistically significant antibacterial effect compared with the combination treatment of MOI 1 and AME at 750 mg/kg for 7 days (*p* = 0.003), whereas the other combination regimens also showed highly significant differences (*p* < 0.0001). This suggests that norfloxacin remained highly effective in the lower urinary tract, whereas the combination therapy showed variable but appreciable antibacterial activity.

In contrast, the kidney‐treated groups showed a different pattern of response. The most effective combination regimen did not differ significantly from norfloxacin, indicating comparable antibacterial efficacy in renal tissue. This finding is noteworthy because renal infections may be more challenging to eradicate than bladder infections, potentially due to differences in tissue accessibility and host–pathogen interactions [27]. Therefore, the comparable performance of the combination treatment in the kidney suggests that the phage–AME approach may merit further evaluation as a potential therapeutic alternative or adjunct in ascending urinary tract infections (UTIs).

Overall, these results highlight that treatment efficacy was site‐dependent and should be interpreted separately for bladder and kidney tissues. The stronger effect of norfloxacin in the bladder and the comparable efficacy of the combination therapy in the kidney underscore the importance of evaluating both organs when assessing antimicrobial strategies in animal models of UTI.

In studies using animal models and in personalized applications across various types of infections, bacteriophage‐treated groups showed reduced bacterial load and improved lung lesions [28–32]. Another study reported that intravenous phage administration improved outcomes in mice with *Acinetobacter*‐associated lung infection [33].

In line with our study, Watanabe et al. demonstrated that oral administration of a lytic phage strain (KPP10) in sepsis caused by *P. aeruginosa* significantly protected mice against mortality (survival rates, 66.7% for the phage‐treated group vs. 0% for the saline‐treated control group; *p* < 0.01). Mice treated with phage also had lower numbers of viable *P. aeruginosa* cells in their blood, liver, and spleen [34]. Similarly, phage therapy reduced the number of *Cronobacter* colonies in the kidney by 70%. Higher levels of malondialdehyde were reduced by phage therapy without affecting the antioxidant status. The expression of pro‐inflammatory cytokines, including tumor necrosis factor‐alpha and monocyte chemoattractant protein‐1, increased following infection and was attenuated by phage therapy [35].

Consistent with our study, there was no difference in bladder colonization with *Cronobacter* between the treated and infected groups. This may suggest that the phage kinetics after intraperitoneal administration could be improved by selecting an alternative route of administration. On the other hand, similar to other studies using the transurethral UTI model, the variance, especially in the cultivation analysis, is high [35]. Sarshar et al. observed a significant reduction in bacterial load in the group pretreated with the aqueous extract of *Orthosiphon stamineus* leaves for 7 days, resulting in a reduction below 10^3^ colonies. This demonstrated the effect of the crude extract on bladder and kidney infections [36]. Our previous studies have demonstrated that both phages and AME have antiadhesive effects [8]. Therefore, this reduction may be related to the antiadhesive properties of the extract and the phages; however, this mechanism was not directly tested in the present study.

In the present study, therapeutic efficacy was primarily evaluated based on viable bacterial counts in the kidney and bladder, which represent a widely accepted indicator of antimicrobial effectiveness in UTI models. No mortality or overt clinical signs of toxicity were observed in any of the treated animals throughout the experimental period, indicating that the administered doses of AME and phage were generally well tolerated. Nevertheless, histopathological examination of infected organs was not performed because both kidneys were homogenized for bacterial counting, which limits the comprehensive assessment of tissue‐level safety and therapeutic impact. Future studies incorporating detailed histopathological and toxicity analyses are, therefore, warranted to further validate the safety profile and therapeutic potential of AME–phage therapy.

Further research is needed to explore the mechanisms underlying these differences in treatment efficacy. Additionally, larger trials could provide more robust data on the optimal dosages and treatment durations, facilitating the development of standardized treatment protocols for future clinical practice.

By continuing to investigate the interplay among dosage, duration, and treatment outcomes, future studies can further enhance our understanding of how best to manage kidney and bladder health through targeted therapies.

## 5. Conclusion

In conclusion, the present findings suggest that the AME–phage combination may represent a promising antimicrobial strategy against *P. mirabilis* infection, particularly in the context of biofilm‐associated UTIs. The observed reduction in bacterial burden indicates that the combination treatment has potential therapeutic value; however, the underlying mechanisms of interaction require further investigation. In addition, the efficacy of this approach appears to depend on treatment dose, duration, and target tissue, underscoring the need for further optimization. Future studies should, therefore, focus on validating these findings in additional infection models, and on addressing physiological barriers that may influence treatment performance before clinical translation can be considered.

## Funding

This work was supported by Isfahan University of Medical Sciences, Isfahan, I.R. Iran, through Grant No. 1401268.

## Ethics Statement

The study was conducted at Isfahan University of Medical Sciences in compliance with international guidelines for animal research and was approved by the local ethics committee (permit number: IR.MUI.AEC.1401.045).

## Conflicts of Interest

The authors declare no conflicts of interest.

## Data Availability

Data sharing is not applicable to this article as no datasets were generated or analyzed during the current study.
